# Revealing the Exciton Fine Structure in Lead Halide Perovskite Nanocrystals

**DOI:** 10.3390/nano11041058

**Published:** 2021-04-20

**Authors:** Lei Hou, Philippe Tamarat, Brahim Lounis

**Affiliations:** 1 Université de Bordeaux, LP2N, F-33405 Talence, France; lei.hou@institutoptique.fr (L.H.); philippe.tamarat@u-bordeaux.fr (P.T.); 2 Institut d’Optique and CNRS, LP2N, F-33405 Talence, France

**Keywords:** lead halide perovskites, nanocrystals, optical spectroscopy, exciton, fine structure, exciton–phonon coupling, biexciton, trion, photon statistics

## Abstract

Lead-halide perovskite nanocrystals (NCs) are attractive nano-building blocks for photovoltaics and optoelectronic devices as well as quantum light sources. Such developments require a better knowledge of the fundamental electronic and optical properties of the band-edge exciton, whose fine structure has long been debated. In this review, we give an overview of recent magneto-optical spectroscopic studies revealing the entire excitonic fine structure and relaxation mechanisms in these materials, using a single-NC approach to get rid of their inhomogeneities in morphology and crystal structure. We highlight the prominent role of the electron-hole exchange interaction in the order and splitting of the bright triplet and dark singlet exciton sublevels and discuss the effects of size, shape anisotropy and dielectric screening on the fine structure. The spectral and temporal manifestations of thermal mixing between bright and dark excitons allows extracting the specific nature and strength of the exciton–phonon coupling, which provides an explanation for their remarkably bright photoluminescence at low temperature although the ground exciton state is optically inactive. We also decipher the spectroscopic characteristics of other charge complexes whose recombination contributes to photoluminescence. With the rich knowledge gained from these experiments, we provide some perspectives on perovskite NCs as quantum light sources.

## 1. Introduction

Since the pioneering works of the group of Kovalenko [1,2] on facile synthesis of colloidal lead-halide perovskite NCs with precise sizes and composition control, the field of research on these materials has grown rapidly in recent years. Outstanding properties of perovskite nanomaterials manifest in many aspects, such as large absorption cross sections [3,4], high luminescence yields [1], large charge carrier mobility and generation efficiency [5], facile synthesis and morphological control [6,7], wide tunability in band gap energy [1,8] and quantum signatures in their light emission [4,9]. A better understanding of those fundamental properties is of prime importance for the development of perovskite NC-based applications in nanophotonics [10,11,12,13,14] and quantum optics [15,16]. This requires a thorough examination of the band-edge exciton fine structure, exciton relaxation mechanisms and the characteristics of the exciton–phonon coupling.

Compared with ensemble measurements, single-particle spectroscopy has become a popular and valuable tool to extract the intrinsic photophysical properties of quantum emitters and their interaction with their microscopic environment, since it allows getting rid of averaging effects over the heterogeneities and temporal dynamics of the nano-emitters within a sample [17]. The use of cryogenic temperatures is particularly appealing since the absorption and emission peaks of single semiconductor quantum dots become extremely narrow, due to the dramatic reduction of thermal dephasing. This allows direct access to the structure of the energy levels and the nature of the charge carriers involved in their photoluminescence (PL). For instance, low temperature single-NC spectroscopy methods have been successfully applied to CdSe NCs to identify their lowest-energy exciton states [18,19,20], unveil their quantum optical properties [21,22], and find their limitations associated with residual dephasing and spectral diffusion [23,24].

For lead halide perovskite NCs, the electronic band structure is mainly governed by the atomic orbitals of lead (Pb) and halide (Cl, Br or I) atoms [25,26,27,28]. The valence band maximum stems from hybridized Pb s-orbitals and halide p-orbitals and has an s-like symmetry with the total angular momentum J^h^ = S^h^ = 1/2. The conduction band is built from the p-orbitals of Pb atoms and undergoes a strong spin–orbit coupling that splits the conduction band into two manifolds, with the lower-energy one having a total angular momentum J^e^ = 1/2. Thus, the low-temperature emission of perovskite NCs arises from the recombination of the band-edge exciton formed by coulombic interaction of a hole with J^h^ = 1/2 and an electron with J^e^ = 1/2. Due to the electron-hole exchange interaction, the band-edge exciton of an NC with a cubic shape and a cubic crystal structure is split into a low-lying dark singlet state with total angular momentum J = 0 and a threefold degenerate optically active triplet state with J = 1 and projection J_z_ = 0, ±1 along the z-axis. For lower symmetry crystal structures or NC shapes [29] the degeneracy of the bright triplet will be further lifted, giving rise to a rich band-edge exciton fine structure with up to four sublevels [30,31]. In the early low-temperature spectroscopic investigations of cesium lead halide single NCs [30,32], the spectral signatures of the bright triplet have been evidenced together with a remarkable brightness of these NCs. It has thus been anticipated that a large Rashba effect due to symmetry breaking of the polar lattice could take place in these materials [33,34,35], split further their fine structure sublevels and reverse the singlet–triplet ordering [33,35]. Such a symmetry breaking could develop due to cation positional instabilities or ionic discontinuities at the NC surface [36], but has never been demonstrated experimentally, making the singlet–triplet ordering of perovskite NCs a subject of debate.

In this review, we aim at outlining recent developments towards the elucidation of the band-edge exciton fine structure and exciton relaxation processes in lead halide perovskite NCs, using a single-NC magneto-optical spectroscopy approach. Magnetic coupling of the fine structure states and Zeeman splittings unveils the entire fine structure, in particular the optically inactive ground singlet state. The distribution of the singlet–triplet energy splittings and the splittings within the bright triplet is discussed with the support of a model of electron-hole exchange interaction. The thermal broadening of the emission line and the evolution of the PL decay with temperature give insights into the nature of the exciton–phonon coupling and the exciton relaxation dynamics. Importantly, they explain why perovskite NCs exhibit a bright photoluminescence at low temperature and in zero magnetic field even though the ground exciton state is dark. We also decipher the properties of other charge complexes, such as trion and biexciton, involved in the photoluminescence of perovskite NCs. Special emphasis will be given to the effect of the ground dark state in the quantum optical properties of the NCs. It stores the exciton for a long lifetime, thus favoring the generation of biexciton and the subsequent cascaded emission of photon pairs. The ubiquity of this behavior in single quantum dots is discussed through a comparison between the emitted photon statistics of perovskite NCs and widely studied chalcogenide NCs. These findings are put into perspective for a potential use of single perovskite NCs as quantum light sources for next-generation quantum technology devices.

## 2. Spectral Structure of the Bright Triplet

**Figure 1:**
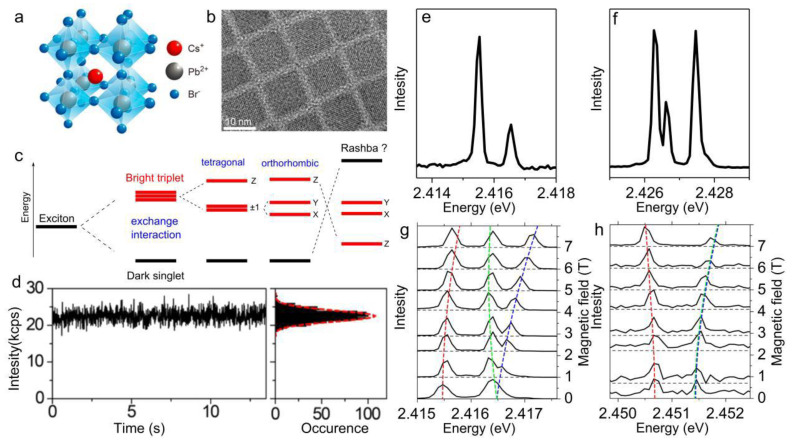
Spectroscopic signatures of the bright triplet exciton. (**a**) Scheme of the crystal structure of CsPbBr_3_ perovskites. (**b**) High-resolution transmission electron microscopy image of CsPbBr_3_ perovskite NCs. (**c**) Energy level diagram of the band-edge exciton fine structure, resulting from electron-hole exchange interaction and crystal field splitting. Radiative recombination of the triplet exciton is electric-dipole allowed (“bright” states), whereas the radiative recombination of the singlet exciton is forbidden (“dark” state). Here we show the evolution of the number of bright sublevels for an NC displaying a single source of anisotropy, either in shape or crystal structure. The hypothetical Rashba effect might reverse the order of triplet and singlet states. (**d**) Time trace of the PL intensity of a single CsPbBr_3_ NC at 2 K. The distribution of photon counts is fitted with a Poisson law (red curve) calculated for an expected value matching the mean number of counts. (**e**,**f**) Examples of low temperature PL spectra of single CsPbBr_3_ NCs, with two lines (**e**) and three lines (**f**). The peaks are the ZPLs associated with the bright exciton fine structure states. (**g**,**h**) Evolution of the triplet spectral structure of two different NCs when applying a magnetic field between 0 to 7 T. The diamagnetic shift is of the order of 4 µeV T^−2^. (**g**) The NC symmetry axis is nearly parallel to the magnetic field. Fitting the Zeeman splitting of the high-energy ZPL yields $g_{∥}^{\mathrm{exc}}$ = 1.9 (**h**). The NC has a symmetry axis nearly perpendicular to the magnetic field. Fitting this evolution leads to $g_{∥}^{\mathrm{exc}}\;$~ 0 and $g_{⊥}^{\mathrm{exc}}\;$~ 2.4. Adapted with permission from ref. [30]. Copyright 2017 American Chemical Society.

Lead halide perovskites of the type APbX_3_, where A is a cation (for example cesium or formamidinium) and X is a halide anion (Cl, Br, or I), are well-known crystals with a crystallographic structure schematically presented in Figure 1a. Lead halide perovskites NCs are synthesized with a cuboid shape [1,2] (see Figure 1b), and then dispersed in a polymer by spin coating on a coverslip, so that their spatial separation allows a study of individual NCs with scanning confocal microscopy. The microscope is operated at cryogenic temperatures to investigate fine structure splittings that are expected in the meV range (see the energy diagram of the band-edge exciton fine structure in Figure 1c). At temperatures of a few Kelvins, the PL intensity of single lead halide perovskite NCs is usually stable, with shot-noise limited variations over minutes of integration time (Figure 1d). Since thermal dephasing is significantly reduced, single NCs of various compositions such as CsPbBr_3_ [4,15,30,33,37,38,39], CsPbI_3_ [32,40,41], FAPbBr_3_ [42,43], FAPbI_3_ [44], MAPbBr_3_ [45] and MAPbI_3_ [46] show extremely sharp lines in their PL spectrum, with line widths often limited by the resolution of the spectrograph (few hundreds of μeV), as shown in Figure 1e,f. These peaks are assigned to zero-phonon radiative recombination lines (ZPLs) of the band-edge neutral exciton. Their spectral structures thus provide the fingerprints of the excitonic triplet sublevels in these NCs.

The triplet spectral structures observed in the single-NC PL spectra offer the opportunity to investigate their differences from one NC to the other. The observation of one, two or three ZPLs is associated with different degrees of degeneracy of the bright triplet, which results from both crystalline structure and NC shape anisotropies (Figure 1c) [29,31,40]. For example, assuming a cubic crystal structure (resp. NC shape), the NC shape (resp. crystal structure) anisotropy will set the number of triplet levels. Spectra with one line, two lines, and three lines will be attributed to a cubic, tetragonal, and orthorhombic anisotropy of the NC shape (resp. crystal structure), respectively. As exemplified in Figure 1e,f, for single CsPbBr_3_ NCs, we observe two categories of PL spectra with either two or three ZPLs [30,38]. Since the orthorhombic crystal structure of bulk CsPbBr_3_ is the most favorable at room temperature [47,48], we can assume that it remains at low temperatures in NCs. The observation of three-ZPL triplet structures is consistent with a nearly cubic morphology, while two-ZPL ones may result from a compensation of the shape and crystal structure anisotropies along a symmetry axis.

Under the effect of externally applied magnetic fields, the triplet sublevels undergo shifts, splitting and coupling, which allow the extraction of the diamagnetic coefficient (a few µeV T^−2^) and the exciton Landé g-factor (Figure 1g,h). Such a determination is straightforward in the case of single CsPbBr_3_ NCs displaying two ZPLs in zero field, which are assigned to $\left|0^{B}\right.\rangle$ and the two-fold degenerate $\left|1^{\pm }\right.\rangle$ triplet sublevels. Two distinct evolutions of the PL spectrum with a magnetic field are observed, depending on the orientation of the NC z-symmetry axis with respect to the magnetic field [30]. As exemplified in Figure 1g, the $\left|1^{\pm }\right.\rangle$ branch may split into two Zeeman components, whose splitting linearly increases with the amplitude of the magnetic field. This situation corresponds to the Faraday configuration, where the NC symmetry axis is oriented along the field. It yields the exciton g-factor (in the parallel configuration) $g_{∥}^{\mathrm{exc}}=g_{∥}^{e}+g_{∥}^{h}$ ~ 2, where $g^{e}$ and $g^{h}$ are respectively the electron and hole g-factors. The NCs showing no Zeeman splitting of the states $\left|1^{\pm }\right.\rangle$ are those that have a symmetry axis perpendicular to the field. Their spectra manifest a magnetic coupling between $\left|0^{B}\right.\rangle$ and $\left(\left|1^{+}\rangle +\right.\left|1^{-}\right.\rangle \right)/\sqrt{2}$ with a coupling strength $g_{⊥}^{\mathrm{exc}}\mu_{B}B/2$, $\mu_{B}$ being the Bohr magneton [49] (Figure 1h). This allows a derivation of $g_{⊥}^{\mathrm{exc}}=g_{⊥}^{e}+g_{⊥}^{h}\approx 2.4$, in accordance with calculations from effective mass models [49]. The derivation of $g^{e}$ and $g^{h}$ for each NC requires magneto-optical investigations of other charge complexes, such as charged excitons, which will be described in Section 5.

## 3. The Dark Ground Exciton State

**Figure 2:**
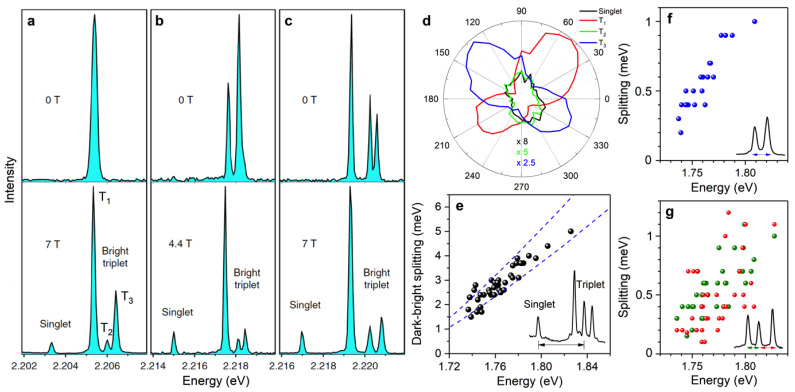
Entire exciton fine structure of single perovskite NCs. (**a**–**c**) Low-temperature PL spectra of three different FAPbBr_3_ NCs displaying one line (**a**), two lines (**b**) and three lines (**c**) in zero field (upper panels). Their conversion under magnetic fields into four-line spectra (lower panels) reveals the entire fine structure, especially the lowest-energy dark singlet exciton state. (**d**) Polarization analysis of the emission lines of the FAPbBr_3_ NC shown in (**a**). The triplet ZPLs are denoted as T_1_, T_2_ and T_3_. (**e**) The splitting between the singlet line and the central triplet line is plotted for many single CsPbI_3_ NCs as a function of the energy of the emitted photons (taken at the central triplet line). The dashed blue lines are simulations of the electron–hole exchange interaction of NCs with a cubic shape, taking into account the two bounds of dielectric corrections associated with the sample boundaries. Top line: NC surrounded by vacuum; bottom line: NC surrounded by sapphire. (**f**,**g**) Zero-field triplet splittings of single CsPbI_3_ NCs with two-line spectra (**f**) and with three-line spectra (**g**) as a function of the energy of the lowest (**f**) or central (**g**) exciton line. Adapted with permission from ref. [42]. Copyright 2019 Springer Nature. Also adapted from ref. [40].

From symmetry considerations of the band edge exciton fine structure, it is predicted that the lowest-energy exciton state of lead halide perovskite NCs is the optically forbidden singlet state labeled $\left|0^{D}\right.\rangle$ with total angular momentum J = 0 [30,49]. Under magnetic fields, this dark exciton should acquire oscillator strength by magnetic coupling with the upper bright triplet sublevels. The component of the magnetic field along the NC symmetry axis is expected to couple $\left|0^{D}\right.\rangle$ to $\left|0^{B}\right.\rangle$ with a strength $(g_{∥}^{e}-g_{∥}^{h})\mu_{B}B/2$, while the perpendicular field component will couple $\left|0^{D}\right.\rangle$ to $\left(\left|1^{+}\rangle -\right.\left|1^{-}\right.\rangle \right)/\sqrt{2}$ with a coupling strength $(g_{⊥}^{e}-g_{⊥}^{h})\mu_{B}B/2$ [49]. Early investigations of the PL decay from ensembles of CsPbBr_3_ perovskite NCs have unveiled the onset of a long-time component under high magnetic fields (above 10 T), pointing to magnetic brightening of a long-lived state attributed to the dark exciton [50,51]. The direct spectroscopic signature of the dark exciton emission under magnetic fields has recently been discovered on hybrid organic–inorganic FAPbBr_3_ and fully inorganic CsPbI_3_ single NCs [40,42], showing evidence for both perovskite species that the ground exciton state is the dark singlet state lying a few meV below the bright triplet in these perovskite NCs (Figure 2a–c). The identification of the bright state that is coupled with the dark state is readily deduced from the polarization of the emission lines, as shown in Figure 2d for a FAPbBr_3_ NC. In this example, the polarization character of the dark state emission line closely follows that of the mid-triplet line, as a signature of an exclusive magnetic coupling between these states. Such analysis, combined with the field dependence of the spectral fingerprints and the PL decay, yields a complete characterization of the entire band-edge exciton fine structure and the relevant Landé g-factors [42].

For both species of perovskite NCs, the dark–bright splitting clearly increases with the energy of the excitonic transition (Figure 2e), as a clear signature of its dependence on the NC size through quantum confinement of the exciton. This shows that singlet–triplet energy separations result essentially from the electron-hole exchange interaction. A theoretical model based on a variational approach has been developed to evaluate the exchange interaction in cuboid shaped NCs. Assuming a cubic crystal structure [40], it takes into account the effects of quantum confinement and dielectric confinement in NCs of various sizes and aspect ratios. It reproduces well the set of singlet–triplet splittings experimentally observed on CsPbI_3_ NCs [40] (Figure 2e), without resorting to the hypothetical Rashba effect. The calculations also show that among all common lead halide perovskite species, CsPbBr_3_ perovskites have the largest bulk electron-hole exchange interaction, which could explain the failed attempts to observe brightening of the optically inactive state in single CsPbBr_3_ NCs with magnetic fields of less than 7 T [30].

The electron-hole exchange interaction also manifests in the triplet splittings [29,31,33,40,52], which are found to decrease with the use of heavier halide atoms in the chemical composition of the NCs [32,33,42]. FAPbI_3_ perovskites have the weakest bulk exchange interaction [40], which is consistent with the observation of a single emission line that likely hides an unresolved exciton fine structure [44]. Moreover, the triplet splittings are also found to increase with the NC emission energy for weekly confined NCs of CsPbI_3_ [40], CsPbBr_3_ [40], FAPbBr_3_ [42] and CsPbBr_2_Cl NCs [33], as exemplified in Figure 2f,g. This trend is the opposite of that predicted for the Rashba effect, whose contribution is expected to decrease with increasing NC sizes [53]. Finally, a strong correlation is observed between the ZPL splittings and the emission energy along single-NC spectral trajectories [40]. This is attributed to fluctuations in the local dielectric environment and further supports the dominant contribution of exchange interaction in the fine structure splittings. A thorough comparison between theory and experiment will require models that take into account both crystal phase and NC morphology anisotropies in the evaluation of the exchange interaction [29], as well as the experimental determination of the crystal structure, shape, orientation, and dielectric local environment of each NC, which is currently far from reach.

## 4. Exciton Relaxation Dynamics and Coupling to Phonons

**Figure 3:**
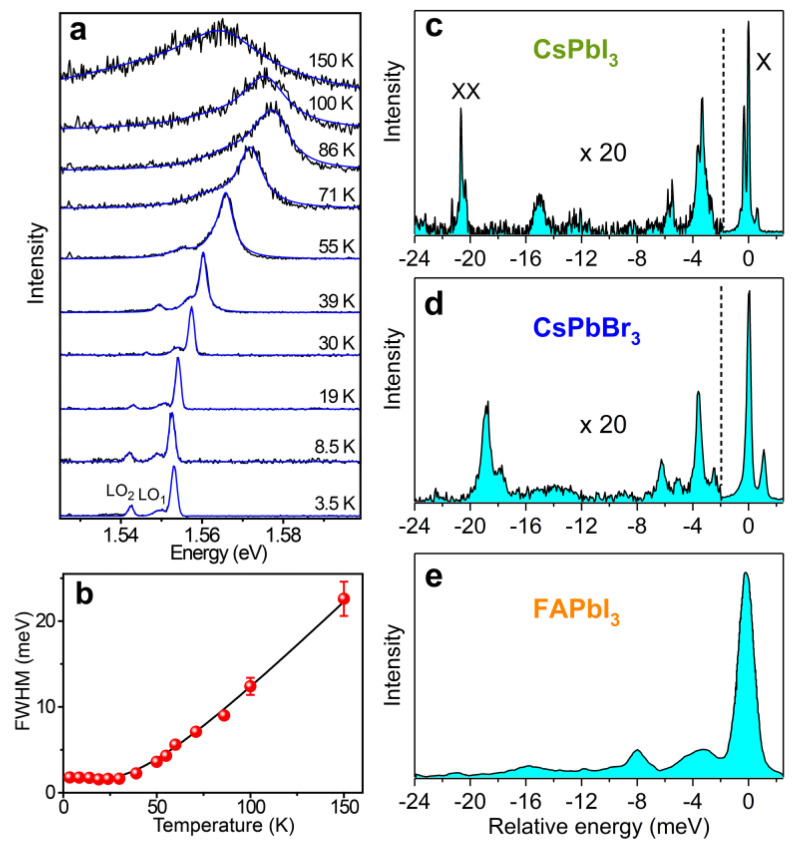
Exciton–phonon coupling. (**a**) Temperature dependence of the PL spectrum of a FAPbI_3_ NC. The spectra are fitted with Gaussian profiles below 30 K and Lorentzian profiles at higher temperatures (blue curves). The spectrum shifts to the blue as the temperature is raised up to ~100 K. This typical behavior of perovskites is attributed to a lowering of the valence band maximum accompanying the lattice expansion [61]. At higher temperatures, non-monotonic shifts of the PL spectrum are usually assigned to phase transitions in the crystalline structures [43,45,55]. (**b**) Evolution of the ZPL line width (FWHM) with temperature for the same NC. The black curve is a fit with the expression of $\Gamma \left(T\right)$, taking $\Gamma_{0}\;$= 1.5 meV, $\gamma_{ac}$ < 5 µeV K^−1^, $\Gamma_{\mathrm{LO}}\;$= 27 meV, $E_{\mathrm{LO}}$ = 10.7 meV. (**c**–**e**) Three main LO phonon replicas are observed in the PL spectra of single perovskite NCs of various compositions: (c) CsPbI_3_, (**d**) CsPbBr_3_ and (**e**) FAPbI_3_ NCs. Note that the structure labeled XX in (**c**) is attributed to the biexciton recombination. Adapted from refs. [40,44].

Despite the presence of an optically inactive ground exciton state, perovskite NCs can remain strikingly bright in zero field and at liquid helium temperature, where the thermal energy is much lower than the dark–bright splitting. An explanation for this behavior requires elucidating the exciton recombination dynamics and the relaxation rates within fine structure sublevels, which are set by the nature and strength of the exciton–phonon coupling. Such coupling manifests through the evolution of the exciton recombination line width with temperature. While spectroscopic studies of perovskite bulk samples [54,55] or ensembles of NCs [56,57] are spoiled by inhomogeneous line broadening, studies of individual NCs provide direct access to the homogeneous line as a function of temperature, as exemplified in Figure 3a for a FAPbI_3_ NC. The contributions of acoustic and optical phonons to thermal broadening of the homogeneous line are readily derived from a phenomenological model initially used for inorganic quantum wells [58], then extended to perovskites [59]. In this model, the contribution of optical phonons is proportional to their Bose–Einstein number, whereas a linear dependence on temperature is assumed for acoustic phonons. As displayed in Figure 3b, homogeneous broadening as a function of temperature T is thus fitted with the expression $\Gamma \left(T\right)=\Gamma_{0}+\sigma_{\mathrm{Ac}}T+\Gamma_{\mathrm{LO}}/\left[\exp\left(E_{\mathrm{LO}}/k_{B}T\right)-1\right]$, where $\Gamma_{0}$ is the zero-temperature line width, $\sigma_{\mathrm{Ac}}$ and $\Gamma_{\mathrm{LO}}$ are the coefficients of exciton–acoustic phonon and exciton–optical phonon interactions, respectively, and $E_{\mathrm{LO}}$ denotes the optical phonon energy. The exciton–phonon coupling coefficients extracted from such fits are listed in Table 1 for single NCs of various compositions. While the contribution of acoustic phonons should dominate at low temperature, it turns out to be negligible, with extremely weak values of $\gamma_{ac}$ for all of the listed perovskite NCs. In the example of Figure 3a, no line width broadening is observed below 30 K and the fit can be performed with $\sigma_{Ac}=0$. Indeed, carrier–acoustic phonon interactions via deformation potentials are impeded due to the strong lattice anharmonicity inherent to these structurally soft materials [60]. Therefore, the room-temperature homogeneous line width is essentially set by the Fröhlich interaction between the exciton and longitudinal optical (LO) phonons. A single LO phonon mode is used to reproduce the thermal broadening of the ZPL, while several polar LO phonon replica of the ZPL show up in the low-temperature PL spectra of single lead halide perovskite NCs, as shown in Figure 3c–e. The $E_{\mathrm{LO}}$ parameter is thus an effective energy in the carrier–LO phonon interaction.

In Table 1, the spectral positions of the most pronounced LO phonon replicas are also listed for various compositions of perovskite NCs. Besides these LO peaks, the large sidebands appearing in the PL spectra of Figure 3c,d are attributed to packets of finely spaced low-energy lattice modes involving the PbX_3_ network vibrations (stretching and bending) coupled to the cation motion. Interestingly, the sets of phonon replicas of Figure 3c,d are very similar within a scale factor of ~1.25 that matches the ratio of square roots of the atomic masses of iodide and bromide atoms, which is consistent with LO phonon modes associated with vibrations of the lead halide network. Yet, significant differences show up with the LO phonon spectrum of FAPbI_3_ NCs (Figure 3e), which indicates that the PbX_3_ network vibrations are strongly influenced by the rigid-body motion of a large cation such as formamidinium. A thorough determination of all phonon modes would require combining these findings with the results derived from infrared absorption spectroscopy [62], Raman spectroscopy and neutron scattering [63].

**Figure 4:**
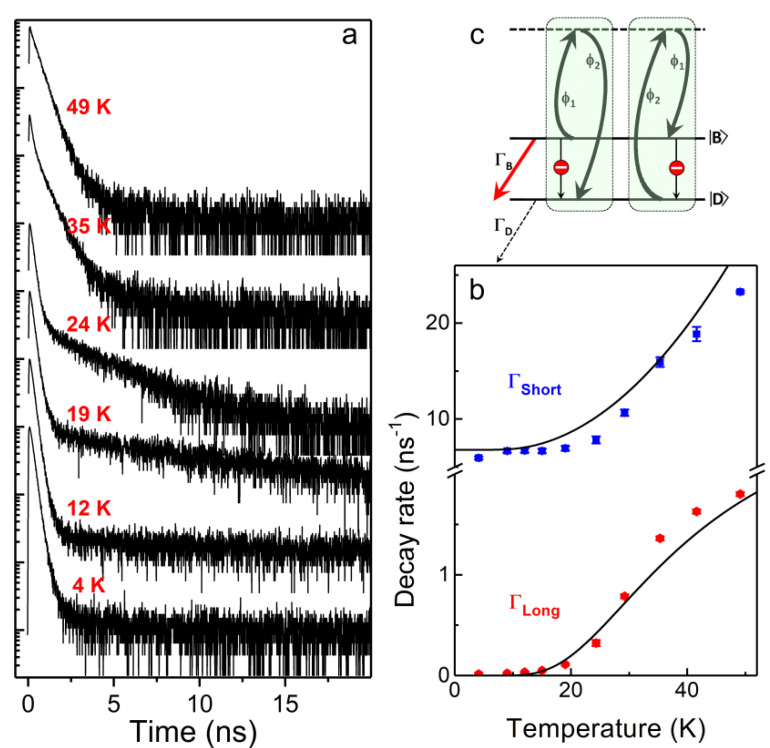
Exciton relaxation dynamics. (**a**) Evolution of the PL decay of a single FAPbBr_3_ NC with temperature. (**b**) Temperature dependence of the short component decay rate $\Gamma_{\mathrm{Short}}$ and the long component decay rate $\Gamma_{\mathrm{Long}}$ extracted from the biexponential fit of the decay curves in (**a**), with 8 ns^−1^, $\Gamma_{D}$ = 10^−4^ ns^−1^, $\gamma_{0\;}$= 31 ns^−1^, $E_{\phi_{1}}$ = 5 meV and $E_{\phi_{2}}$ = 8.4 meV. (**c**) Scheme of the two-phonon thermal mixing model between the dark state $\left|D\right.\rangle$ and one bright state $\left|B\right.\rangle$ with relaxation rates $\Gamma_{D}$ and $\Gamma_{B}$, respectively. The red stop disk indicates that bright-to-dark relaxation with a one-phonon process is inhibited in lead halide perovskites NCs. Adapted with permission Scheme is from ref. [42]. Copyright 2019 Springer Nature.

The recombination dynamics of the exciton and the associated relaxation rates within the fine structure sublevels can be extracted from the evolution of the PL decay of single NCs with temperature. For FAPbI_3_, FAPbBr_3_ and CsPbI_3_ perovskite single NCs, the PL decay is monoexponential with a sub-nanosecond lifetime at liquid helium temperature, and it becomes biexponential as the temperature is raised, as shown in Figure 4a. The long component of the decay gains weight and shortens with temperature, whereas the short component shortens and disappears above 70 K. This evolution is characteristic of thermal mixing between the long-lived singlet state and the bright states. Strikingly, it cannot be reproduced using the conventional one-phonon thermal mixing model successfully used for CdSe NCs [67]. This results from the lack of exciton–acoustic phonon interaction in perovskites. It also points to a reduced Rashba-like spin–orbit coupling [68,69,70] that would enable one-phonon spin-flip from bright to dark sublevels. Instead, for all of these perovskite NCs, the evolution of the PL decay with temperature is well reproduced with a two-phonon thermal mixing model between the dark state and a bright state based on the absorption and emission of phonons $\phi_{i}$ (i=1, 2) whose energy difference matches the dark–bright splitting [40,42,44] (Figure 4b,c). In this model, the uphill and downhill transition rates between these sublevels are respectively $\gamma_{↑}=\gamma_{0}N_{\phi_{2}}\left(N_{\phi_{1}}+1\right)$ and $\gamma_{↓}=\gamma_{0}N_{\phi_{1}}\left(N_{\phi_{2}}+1\right)$, where $N_{\phi_{i}}=1/\left[\exp\left(E_{\phi_{i}}/k_{B}T\right)-1\right]$ are the Bose–Einstein phonon numbers and $\gamma_{0}$ is a characteristic two-phonon mixing rate. As a consequence of thermal mixing with higher-order phonon processes, the non-radiative bright-to-dark relaxation rate vanishes at low temperatures, which explains why lead halide perovskite NCs exhibit a bright photoluminescence in the form of pure triplet emission despite the fact that the lowest-energy exciton state is optically inactive. This unique property of perovskite NCs makes them promising for a potential use as bright quantum light sources at low temperatures.

## 5. Other Charge Complexes

**Figure 5:**
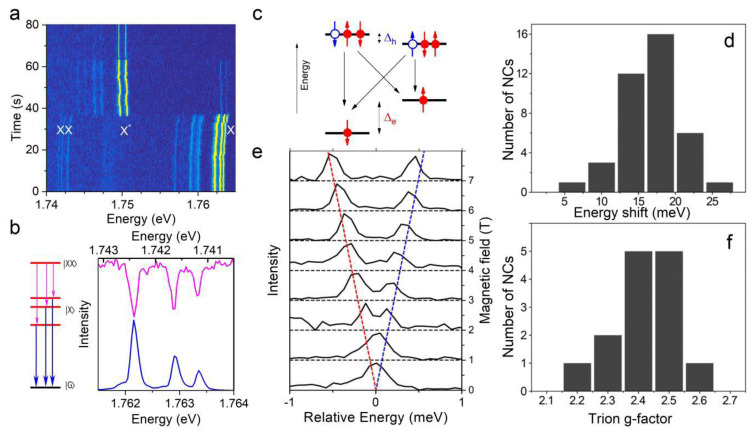
Biexciton and trion spectral signatures. (**a**) Spectral trajectory of a single CsPbI_3_ NC. After 38 s, it switches between exciton (X) emission, together with biexciton (XX) emission, and trion (X*) emission. The correspondence between the biexciton spectral structure and the exciton multiplet is evidenced in (**b**). (**c**) Scheme of energy levels and recombination transitions for a negative trion under magnetic fields. The level splitting Δ_h_ associated with the hole is smaller than that associated with the electron Δ_e_. The red (blue) arrows with solid (hollow) circles represent the spin states of electrons (holes). (**d**) Histogram of the red spectral shifts of the X* structure with respect to X, for 39 single CsPbBr_3_ NCs. (**e**) Zeeman splitting of the X* emission line, yielding a trion g-factor of 2.4. (**f**) Histogram of trion g-factors for 14 CsPbBr_3_ NCs. Adapted with permission from ref. [30]. Copyright 2017 American Chemical Society. Also adapted from ref. [40].

At elevated excitation intensities, another charge complex that is commonly observed in perovskite single NC PL spectra is the biexciton, which consists of two electron-hole pairs in the NC. A biexciton can form at high excitation rates when the NC absorbs two photons simultaneously [71]. In the early ensemble studies of perovskite NC films or colloidal solutions at room temperature, the formation of biexciton was inferred from the ultrashort lifetime (tens of picoseconds) observed in the PL decay and pump-probe transient absorption measurements [71,72]. The spectral signature of biexciton has been revealed in the low-temperature PL spectra of single CsPbI_3_ NCs [32,40], as shown in Figure 5. The spectral trail displayed in Figure 5a over the first 38 s of acquisition shows the spectral fingerprint of an NC in its neutral state, where the ZPLs labeled X for the exciton and XX for the biexciton show up. A signature of the biexcitonic nature of the XX structure comes from the super-linear dependence of its amplitude on the excitation intensity [35,39]. The attribution of these spectral structures is further evidenced by their perfect match when mapping the mirror image of the XX multiplet onto the X one (Figure 5b). This also allows a direct correspondence between the transition lines of the X and XX multiplets. The binding energy of the biexciton can be evaluated from the energy separation between XX and X structures, and is distributed in the range of 10–20 meV for weakly confined CsPbI_3_ NCs [35,39]. Interestingly, identical relative weights between the corresponding XX and X lines show evidence for extremely slow phonon relaxation within the triplet sublevels, with a characteristic time longer than the exciton recombination lifetime. The biexciton lifetime is ~400 ps [32], which is slightly shorter than the neutral exciton lifetime, indicating that non-radiative Auger recombination moderately contributes to the biexciton decay process [73]. This property is appealing for the generation of cascaded photon pairs on the $\left|\mathrm{XX}\right.\rangle \to \left|X\right.\rangle$ and $\left|X\right.\rangle \to \left|G\right.\rangle$ transitions (Figure 5b).

As observed in the spectral trail of Figure 5a, most of the lead halide perovskite NCs occasionally exhibit emission switches between (X, XX) ZPL multiplets and a single redshifted peak named X* attributed to the recombination ZPL of a charged exciton, i.e., a trion. A singly charged exciton can indeed form in an NC when an exciton is created in the presence of an unpaired charge carrier in the core and a charge carrier trapped at its surface or at a lattice defect. It thus consists of two electrons bound to a hole (negatively charged trion) or two holes bound to an electron (positively charged trion). Due to pairing of the two electrons (resp. two holes), the lowest-energy states of a trion form a doublet having a total angular momentum set by that of the unpaired hole (resp. electron) (Figure 5c). This results in the absence of electron-hole exchange within this charge complex and explains why the trion emission is characterized by a single ZPL in the absence of magnetic field.

The trion binding energy can be readily evaluated from the energy separation between X* and X spectral structures and is found in the range of 5–25 meV for inorganic or hybrid single perovskite NCs investigated so far [23,31,36,37,39] (Figure 5d). The count rates of the exciton and trion emissions are similar in perovskite NCs, which indicates that the quantum yields of these two charge complexes are comparable. This points to a moderate participation of non-radiative Auger recombination processes in the trion recombination. The trion may be stable for an extended observation time, from minutes to hours [30], making these materials a promising platform for quantum spin technologies. For all studied NCs, the trion decay is mono-exponential with a lifetime in the sub-ns range, close to half of the exciton lifetime [9,30,33,37,74,75]. Since the radiative decay rate of the trion is expected to be twice faster than that of the neutral exciton [76], these results confirm the modest contribution the non-radiative Auger processes in the trion recombination.

Magneto-optical spectroscopy of charged NCs gives direct access to the spin properties of individual charge carriers, in particular to $g^{e}$ and $g^{h}$. Indeed, the angular momentum of the ground state is set by the charge carrier, while that of the trion state stems from the opposite charge carrier (Figure 5c). In general, the trion recombination under a magnetic field of amplitude B presents four transitions that are shifted in energy by $\left(\pm g^{e}\pm g^{h}\right)\mu_{B}B$ from the zero-field line [77], as depicted in Figure 5c. The magneto-optical investigations of the trion PL in single CsPbBr_3_ [30], CsPbI_3_ [40] and FAPbBr_3_ [42] demonstrate that the trion line always splits into two Zeeman components, as exemplified in Figure 5e. The distribution of the trion recombination g-factors is peaked around 2.4 (Figure 5f), 2.3 and 2.6 for these materials, respectively. These narrow distributions indicate that the magnetic response of the charged perovskite NCs is nearly isotropic. Altogether, the measurements of exciton and trion Landé factors lead to the conclusion that $g^{e}$ approaches that of free electrons ($g^{e}$ ≈ 2), while $g^{h}$ is much smaller ($g^{h}$ ≲ 0.4) in these perovskites [30].

## 6. Perovskite Nanocrystals as Quantum Light Sources

**Figure 6:**
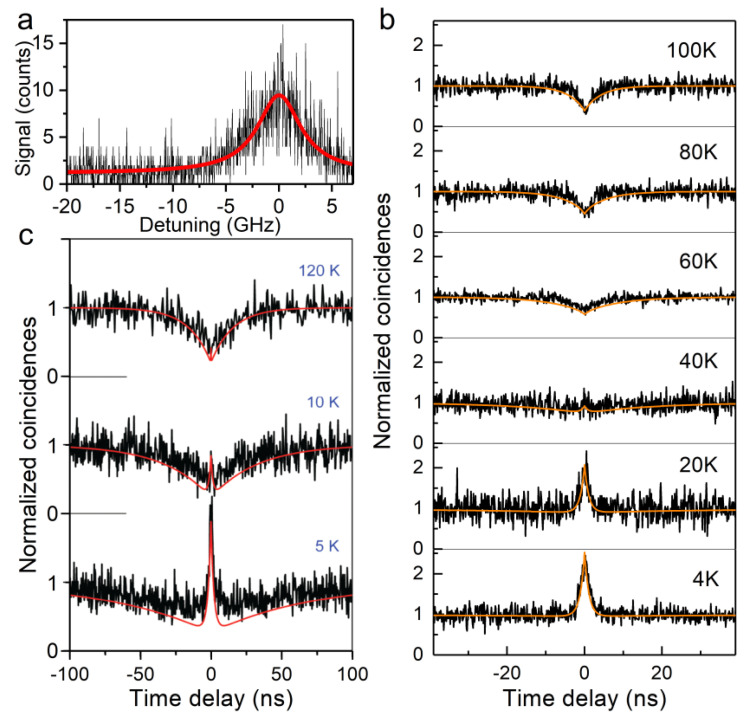
Tunable photon statistics of single quantum dots. (**a**) Resonant PL excitation spectrum of a single CsPbI_3_ NC. It is fitted with a Lorentzian profile with a line width of 5 GHz FWHM (red curve). (**b**,**c**) Normalized photon coincidence histograms of a single CsPbI_3_ NC (**b**) and a CdSe NC (**c**) at various temperatures, showing a similar conversion from bunching to antibunching as the dark and bright exciton sublevels become thermal mixed. The red curves are numerical simulations of the PL autocorrelation function on the basis of a four-level model [40]. Adapted from ref. [40].

The bright PL of perovskite NCs, associated with a reduced thermal broadening of the homogenous lines at cryogenic temperature, makes perovskite NCs attractive solid-state emitters for potential use as quantum light sources. Notably, sources of single indistinguishable photons on-demand are needed in various quantum information processing schemes [78]. Emitted photons become indistinguishable when the optical coherence lifetime of the quantum emitter $T_{2}$ approaches twice the lifetime $T_{1}$ of the emitting state. The achievement of the indistinguishability character in solid-state emitters is challenging due to optical decoherence processes induced by exciton–bath interactions, such as phonon-scattering, spin-noise and charge density fluctuations in their environment, often leading to $T_{2}\ll 2T_{1}$. In order to probe the optical coherence lifetime of single perovskite NCs, methods based on the first-order correlation function of the emitted light (Fourier spectroscopy) [79,80] led to $T_{2}$ ranging from 50 to 80 ps for single CsPbBr_3_ NCs, which translates into $T_{2}/2T_{1}$ up to 0.2 and $T_{2}\;$~ 76 ps for single CsPbI_3_ NCs, corresponding to $T_{2}/2T_{1}\;$~ 0.04 [41]. With these methods, the excess of energy between absorption and emitted photons may generate spectral diffusion of the emission transition [42]. An alternative method to extract the ultimate limit of $T_{2}$ relies on the resonant excitation of single quantum dots on their excitonic ZPLs [81,82,83]. It consists in sweeping the frequency of a single-mode CW laser across the ZPL while collecting the red-shifted signal emitted into the phonon replicas. Using this method, a homogeneous line width $1/\pi T_{2}$ ~ 5 GHz FWHM has been observed for one of the triplet exciton ZPLs (Figure 6a), corresponding to $T_{2}$ ~ 64 ps [40]. This value is still significantly shorter than the exciton lifetime $T_{1}$ ~ 1 ns, as a result of residual dephasing processes. The latter are likely caused by fluctuations in the local electric field and dielectric screening upon charge displacements at the NC surface or in its nano-environment.

Interestingly, photon correlation measurements on individual perovskite NCs demonstrate that the dark exciton ground state favors the creation of biexcitons at low temperatures and thus the emission of correlated photon pairs. Experimentally, a Hanbury Brown and Twiss coincidence setup is used to build the histogram of time delays between consecutive photons emitted by the NC. Strong photon bunching is found for all single CsPbI_3_ NCs at 4K, as a hallmark of multiphoton emission. This feature is attributed to the presence of a long-lived ground exciton state, which shelves the exciton and thus favors the generation of biexcitons [35]. Radiative relaxation induces a cascaded photon emission on the biexciton–triplet exciton and triplet exciton–zero exciton transitions. Following the evolution of the coincidence histograms of single NCs as the temperature is raised, photon bunching clearly converts to photon antibunching above 60 K (Figure 6b). This behavior is the manifestation of the vanishing exciton-shelving action played by the long-lived exciton level as dark–bright thermal mixing operates. It is well reproduced with simulations of the autocorrelation function derived from the solutions of rate equations in a four-level model taking into account the biexciton, thermally mixed bright and dark levels, and the ground zero-exciton level. In the same vein, magnetic coupling of the dark and bright exciton states was found to weaken the degree of photon bunching, as it shortens the long component of the PL decay [35].

The promotion of correlated photon pairs by a long-lived ground exciton level in quantum dots seems to be ubiquitous, since similar temperature-dependent photon statistics were observed on single CdSe NCs [35] (Figure 6c), which have a long-lived ground exciton level with total angular momentum projections $\left|2^{\pm }\right.\rangle$ located ~2 meV below the lowest short-lived bright level $\left|1_{L}^{\pm }\right.\rangle$ [19]. Compared with the case of perovskite NCs, the conversion from bunching to antibunching occurs at much lower temperatures (below 10 K), as soon as the thermal energy approaches the energy splitting between the long-lived and short-lived sublevels. Indeed, thermal mixing is mediated in CdSe NCs by a single acoustic phonon whose energy matches these level splittings [67,82,84], while in perovskites it develops through a second-order process with higher-energy phonons. Another difference is that while the ground singlet of perovskites is optically inactive, the ground long-lived excitonic level of CdSe NCs has a radiative recombination that is evidenced by a dominant ZPL in the PL spectra and a dominant long component in the PL decay at 2 K and in a zero magnetic field [84,85]. The radiative yield of the ground exciton in CdSe NCs has been attributed to its coupling with the higher-energy bright excitons through an effective internal magnetic field caused by dangling-bond spins at the NC surface [85,86,87]. Thus, the long-lived ground exciton state of single quantum dots has the same effect in their photon statistics, regardless of its quantum yield.

## 7. Conclusions and Outlook

Using magneto-optical spectroscopy of single perovskite NCs at cryogenic temperatures, a wealth of information has been unraveled on the optical properties of their exciton and other charges complexes. The band-edge exciton fine structure of inorganic as well as hybrid lead halide perovskite NCs consists of a bright triplet and a ground dark singlet located several meV below as a result of electron-hole exchange interaction, which is enhanced by the combined effects of quantum and dielectric confinements. This closes the debate concerning the relative order of dark and bright sublevels in these materials. The energy splittings within the fine structure also result from a subtle interplay of crystal structure and shape anisotropy effects. The temperature dependence of the homogeneous broadening reveals an extremely reduced exciton–acoustic phonon coupling, which is of prime importance for the development of next-generation devices for photovoltaics since the intrinsic limits to the mobility of charge carriers are fundamentally set by phonon scattering. These spectroscopic findings are consistent with a phonon glass state in these soft materials, where strong lattice anharmonicity hinders electron scattering by acoustic phonons. They will help elucidate the hot phonon bottleneck that affects the charge carrier transport in perovskites. Additionally, the thorough characterization of the band-edge exciton properties is essential for a better understanding of the strong light–matter interaction regimes [88]. A key property of perovskite NCs that makes them suitable for potential use as bright quantum light sources at liquid helium temperature lies in their inhibited bright-to-dark relaxation. Indeed, such relaxation requires a Raman-like two-phonon process that vanishes at low temperatures. The protection of triplet states against spin relaxation should also promote new applications of perovskites in spin-based technologies. While bright-to-dark relaxation is forbidden at low temperature, the long-lived ground exciton plays a major role in the quantum optical properties of the NCs, by favoring the formation of a biexciton and thus the emission of correlated photon pairs. This makes perovskite NCs appealing solid-state quantum emitters with tunable photon statistics controlled by temperature or magnetic field. Further efforts will be devoted to the reduction of the dephasing rate in these materials in order to produce indistinguishable photons, for instance through the application of strong local electric fields that could stabilize the charge distributions in the NC, or through enhanced surface passivation with suitable shells or ligands. The realization of ideal sources of entangled photons will require degenerate bright triplet emission that may be achieved in NCs with specific morphologies and crystal structures and/or with the application of external fields. The next step will be to investigate the quantum optical properties of the photoluminescence stemming from lead halide nanocrystals that are self-organized into three-dimensional superlattices [16]. These mesoscopic structures could indeed be optimized in terms of interactions between emitters and decoupling from their environment, so that they exhibit a large acceleration of the radiative decay and strong photon bunching. Such entangled multi-photon quantum light sources should favor the inception of next-generation quantum technology devices.

## Figures and Tables

**Table 1:** Energies of the three main LO phonon replicas and exciton–phonon coupling parameters of several lead halide perovskite NCs. *R_Bohr_* is the exciton Bohr radius; $E_{g}^{Bulk}$ is the bulk bandgap energy; *ε_eff_* is the effective dielectric constant; L is the NC size; LO_1_, LO_2_ and LO_3_ are the main phonon peaks observed in the PL spectra.

NCs	R_Bohr_ (nm)	$E_{g}^{Bulk}$ (eV)	*ε_eff_* [64,65]	L (nm)	Red-Shifts of the Main LO Phonon Sidebands (meV)			Exciton–Phonon Coupling Parameters			
					LO_1_	LO_2_	LO_3_	γ_ac_ (µeV/K)	γ_LO_ (meV)	E_LO_ (meV)	Ref.
CsPbBr_3_	3.06	2.25	~7	5~10	3.7	6.3	18.9	8 ± 3	42 ± 15	16	[30,40,66]
CsPbI_3_	4.64	1.72	~10	11.2 ± 1.2	~3.4	~5.5	15	<8	37	16.7	[40]
FAPbBr_3_	3.87	2.23	8.4	9.2 ± 0.7	4.3 ± 0.5	8.6 ± 0.9	13.2 ± 1.1	5 ± 5	52	15.2	[42,43]
FAPbI_3_	5.49	1.5	9.4	10~15	3.2	7.8	15.4	<5	27	10.7	[44]

## Data Availability

Not applicable.
